# Prognostic value of cardiac biomarkers in COVID-19 infection

**DOI:** 10.1038/s41598-021-84643-6

**Published:** 2021-03-02

**Authors:** Aakash Sheth, Malak Modi, Desiree’ Dawson, Paari Dominic

**Affiliations:** Division of Cardiology, The Department of Medicine and Center of Excellence for Cardiovascular Diseases & Sciences, Louisiana State University Health Sciences Center-Shreveport (LSUHSC-S), 1501 Kings Hwy, Shreveport, LA 71103 USA

**Keywords:** Prognostic markers, Cardiology, SARS-CoV-2

## Abstract

Multiple Biomarkers have recently been shown to be elevated in COVID-19, a respiratory infection with multi-organ dysfunction; however, information regarding the prognostic value of cardiac biomarkers as it relates to disease severity and cardiac injury are inconsistent. The goal of this meta-analysis was to summarize the evidence regarding the prognostic relevance of cardiac biomarkers from data available in published reports. PubMed, Embase and Web of Science were searched from inception through April 2020 for studies comparing median values of cardiac biomarkers in critically ill versus non-critically ill COVID-19 patients, or patients who died versus those who survived. The weighted mean differences (WMD) and 95% confidence interval (CI) between the groups were calculated for each study and combined using a random effects meta-analysis model. The odds ratio (OR) for mortality based on cardiac injury was combined from studies reporting it. Troponin levels were significantly higher in COVID-19 patients who died or were critically ill versus those who were alive or not critically ill (WMD 0.57, 95% CI 0.43–0.70, p < 0.001). Additionally, BNP levels were also significantly higher in patients who died or were critically ill (WMD 0.45, 95% CI − 0.21–0.69, p < 0.001). Cardiac injury was independently associated with significantly increased odds of mortality (OR 6.641, 95% CI 1.26–35.1, p = 0.03). A significant difference in levels of D-dimer was seen in those who died or were critically ill. CK levels were only significantly higher in those who died versus those who were alive (WMD 0.79, 95% CI 0.25–1.33, p = 0.004). Cardiac biomarkers add prognostic value to the determination of the severity of COVID-19 and can predict mortality.

## Introduction

The Coronavirus disease 2019 (COVID-19), caused by Severe Acute Respiratory Syndrome Coronavirus-2 (SARS-CoV-2), has affected over 2,402,250 people worldwide and caused 163,097 deaths as of April 21, 2020^1^. Lung injury and acute respiratory distress syndrome are the most common presentation of COVID-19, but cardiac injury is another grim consequence of this multisystem viral disease. Evidence of an association between other systemic viral infections such as influenza^2^ and SARS^3^ and cardiovascular adverse events dates back to the 1930s^4^. Early observational studies in China and reports from The Centers for Disease Control and Prevention (CDC) suggest that patients with pre-existing heart conditions are more likely to develop severe illness from COVID-19^5^. A recent study^6^ suggests that patients in the ICU are three-fold more likely to have pre-existing cardiovascular conditions than non-ICU patients. Conversely, newer evidence implies new onset myocardial injury caused by COVID-19^7,8^. Possible mechanisms of myocardial injury caused by COVID-19, termed acute COVID-19 cardiovascular syndrome (ACovCS), include heightened myocardial demand in response to the stress of infection, inflammatory cytokines creating a thrombogenic environment as the result of platelet activation and endothelial dysfunction, and direct myocardial damage^9,10^. However, current evidence linking myocardial injury as measured by cardiac biomarkers to the severity of disease is conflicting, with a few studies^11–13^ suggesting an association but several others showing a lack of association^14,15^. Given the discrepancy among these reports, it is prudent to investigate the association of cardiac biomarkers with the severity of disease experienced by and the survival or death of COVID-19 patients. Here we present a meta-analysis of 16 observational studies from China to shed further light on this topic.

## Methods

Our meta-analysis was carried out in accordance with the recommendations of the Meta-analysis of Observational Studies in the Epidemiology Group (MOOSE)^16^.

### Search strategies

**Figure 1:**
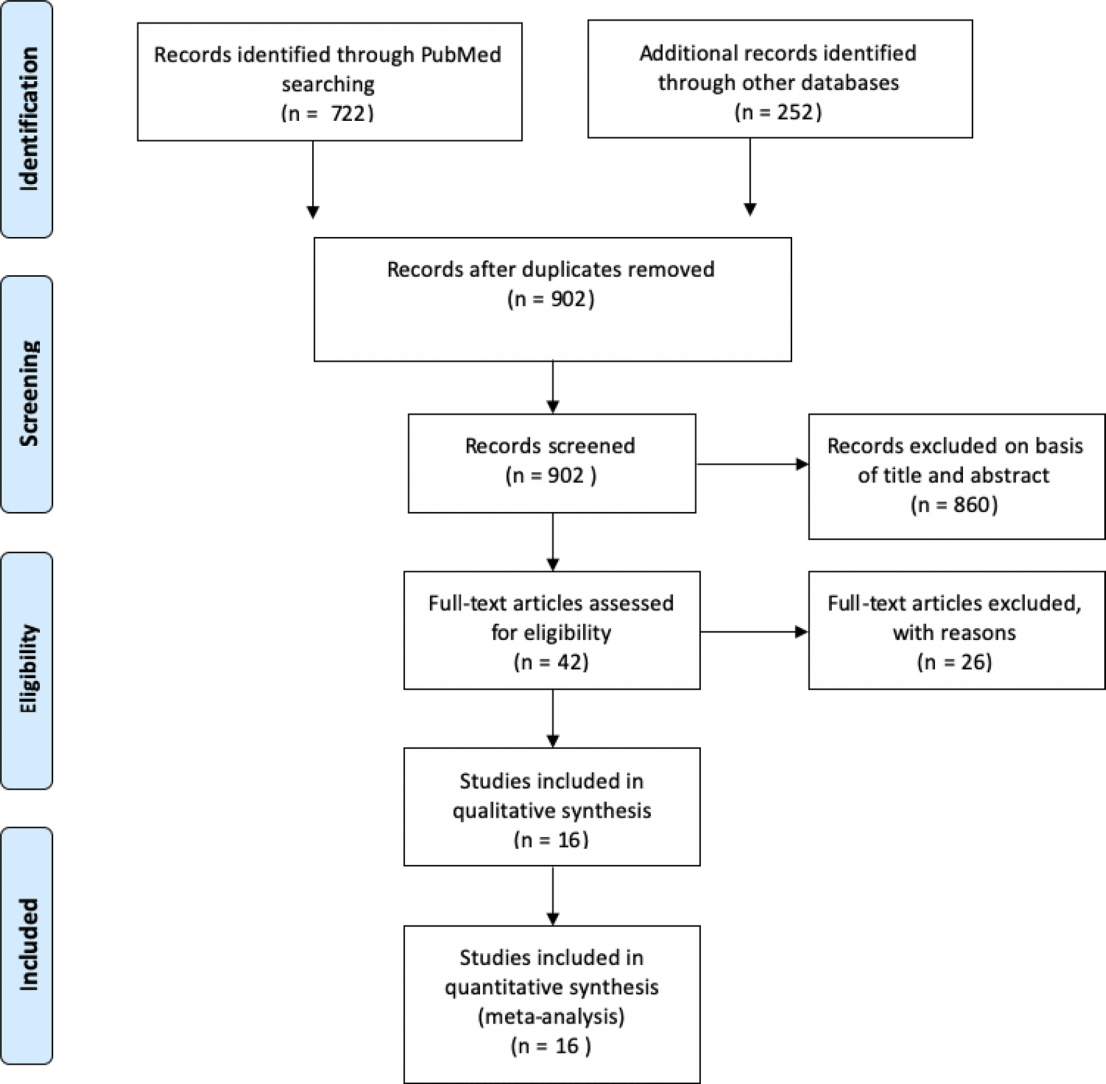
Prisma diagram of literature search and selection.

We searched MEDLINE/PubMed (1966–2020) on 4/15/2020 using the following keywords: ("covid-19" OR "COVID-19" OR "SARS-CoV-2" OR "coronavirus" AND "19") AND (“Clinical Study” OR “Comparative Study” OR “Evaluation Studies” OR “Meta-Analysis” OR “Multicenter Study” OR “Observational Study” OR “Twin Study” OR “Technical Report” OR "cohort studies" OR "cohort" OR "case–control" OR "cross-sectional" OR “observational” OR “comparative” OR "evaluation"). We also used the ‘Related article’ feature on PubMed, along with a manual search of references, to identify additional studies. Further, we performed an additional search on Embase and Web of Science, limited to 4/15/2020 and all searches were finalized on 11/10/2020. We reviewed the full text of all relevant articles. English translations, if necessary, were obtained. Titles and abstracts were independently reviewed by three reviewers (AS, DD, and MM) and cross-verified for inclusion. Details of the search strategy are reported in Fig. 1.

### Inclusion criteria

#### For the analysis of cardiac biomarker levels in patients with COVID-19

Studies (retrospective and prospective) comparing mean or median troponin and/or BNP in COVID-19 patients who were critically ill versus not critically ill, and those who died versus those who survived, were included. Mean or median levels of other biomarkers included in the studies like CK, D-Dimer, LDH, IL-6, and/or CRP levels in COVID-19 patients were analyzed and compared between patients who were critically ill versus not critically ill, and those who died versus those who survived, only when reported in studies that fit our initial inclusion criteria. Adult respiratory distress syndrome was considered a surrogate for critical illness and studies that compared findings in COVID-19 patients who developed ARDS to patients without ARDS were included. As these were in-hospital outcomes, no specific follow up period was set as an inclusion criteria.

#### For the analysis of risk of mortality in COVID-19 patients based on cardiac injury

Studies reporting the risk of mortality with COVID-19 based on the presence of cardiac injury by event rates or univariate or multivariate logistic regression analysis were included. Cardiac injury, for the purpose of this meta-analysis, was defined as an increase in Troponin I, Troponin T, or CK MB. For both groups of studies, the timing of the blood draw during the hospitalization was not a pre-specified inclusion criteria.

### Exclusion criteria

Studies were excluded if they (i) only gave descriptive statistics of the biomarkers in one group of patients with COVID-19, (ii) were published only in abstract form, (iii) were non-English studies with no English translation, and/or (iv) were studies that reported measures of central tendency in groups other than those under consideration, i.e., died versus survived and critically ill versus not critically ill.

### Data extraction and assessment of study quality

For each study included, all data elements reported uniformly across most studies were extracted by two reviewers (AS and MM), cross-verified by a third (DD), and included in Table 1. The quality of each study and the risk of bias were evaluated using the Newcastle–Ottawa Quality Assessment Scale for non-randomized studies^17^. The following characteristics were assessed for sources of bias: (1) patient selection including definitions of exposure and representation of the larger population; (2) comparability of study groups and controlling for confounding factors by design or analysis; and (3) assessment and documentation of outcome including duration and loss of follow-up. Studies were graded as ‘poor’ if they met four or fewer of the nine criteria, ‘fair’ if they met five to six criteria, and ‘good’ if they met more than six criteria. For uniformity, all studies, regardless of study design, underwent quality bias assessment using the retrospective cohort scoring tool.

**Table 1:**
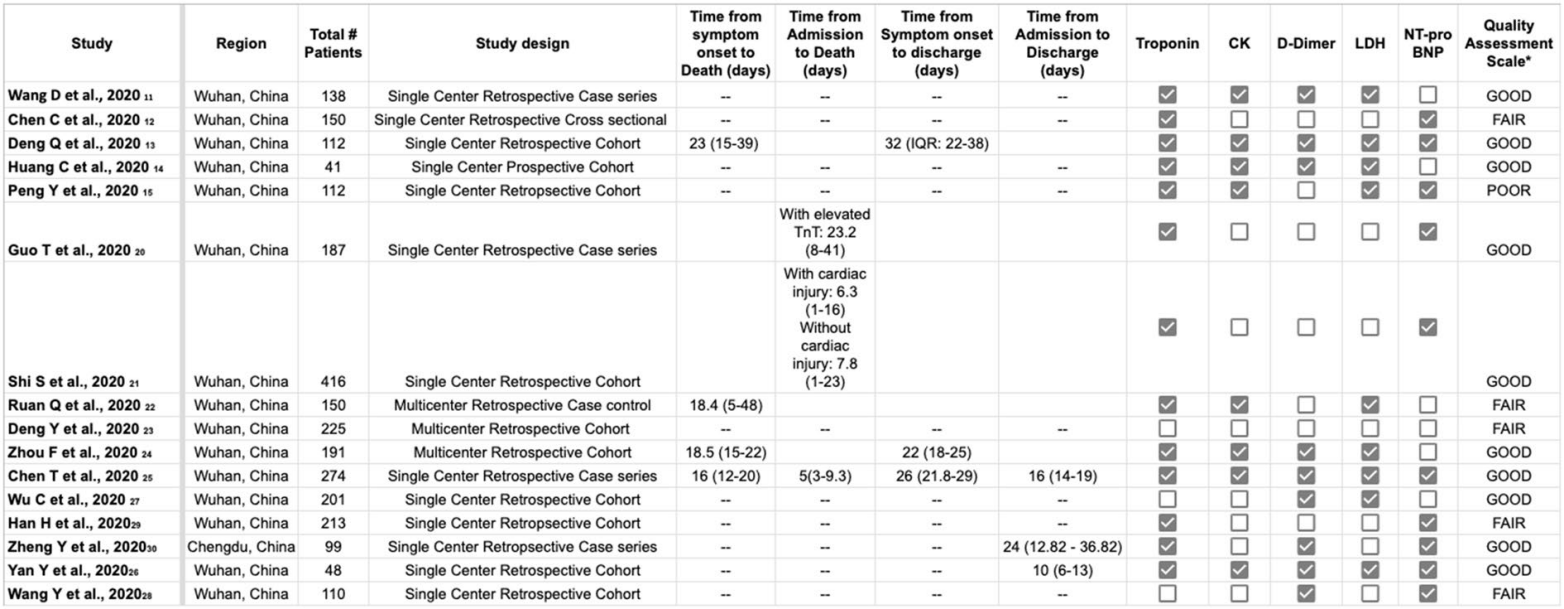
Clinical characteristics and laboratory markers of patients affected by the coronavirus disease 2019 (COVID-19), in the included studies.

*Newcastle–Ottawa Quality Assessment Scale. Poor < 4, Fair 5–6, Good > 7.

### Statistical methods

For the analysis of measures of central tendency of cardiac biomarkers in COVID-19 patients who died or were critically ill, median biomarker values were extracted for patients who died or were critically ill and patients who survived and/or were not critically ill, and the weighted mean differences (WMD) and 95% confidence interval (CI) between these two groups were calculated for each study. For the analysis of the risk of mortality with COVID-19 infection based on cardiac injury, event rates or univariate and multivariate odds ratios (OR) for mortality were extracted. For studies reporting Hazard ratio (HR) only, HR was adopted as the best estimate of OR. OR were transformed logarithmically, then standard error was calculated from Log OR and the corresponding 95% confidence interval (CI). The inverse variance method was used to achieve a weighted estimate of the combined overall effect. For all the analyses, we assessed the results for heterogeneity in our analysis by examining the forest plots and then calculating a Q statistic, which we then compared with the I^2^ index. We considered the presence of significant heterogeneity at the 5% level of significance (for the Q test) and values of I^2^ exceeding 56% as an indicator of significant heterogeneity^18,19^. We adopted the random effect model to pool WMD or effect sizes. All analyses were performed using Comprehensive Meta-Analysis version 3 (Biostat Inc., Englewood, NJ, USA).

For Meta-regression, we adopted a weighted regression random effect model and carried out a multivariate regression of pre-determined factors using comprehensive meta-analysis version 3. These variables were selected based on traditional factors shown to affect cardiovascular health and on data availability for the majority of the studies included. A two-sided P-value < 0.05 was regarded as significant for all analyses. Data were represented as forest plots, and potential publication bias was assessed with the Egger test and represented graphically with Begg funnel plots of the natural log of the OR versus its standard error.

## Results

Sixteen studies with 2667 patients were included in the study. Through the initial database search, 974 studies were investigated for compliance with the inclusion criteria, of which 42 studies were ultimately chosen for consideration. Twenty-six studies were excluded from the meta-analysis for the following reasons: (1) 4 studies were descriptive and only reported biomarkers in all patients and did not compare critical/died versus not critical/alive patients. These studies did not define cardiac injury and did not assess OR for mortality with cardiac injury; (2) 19 studies did not report the biomarkers being studied; (3) 2 studies reported biomarkers based on increased or normal D-dimers; and (4) 1 study did not provide official translation to English. Fifteen studies were retrospective, and thirteen were single center (Table 1). Of the studies that reported time to blood sample collection, the average time reported was at the time of admission to 48 h after admission. Of the studies that reported time to death or discharge, most of the studies reported it as the time from symptom onset to death or discharge. The average time for symptom onset to discharge was 27 days, and from symptom onset to death was 19 days. Two studies did not report median/mean cardiac biomarkers by predetermined patient groups^20,21^, but reported an OR for mortality based on cardiac injury. 5 studies reported biomarkers based on whether the patients survived or died^20,22–26^, 9 studies reported them based on whether the patient was critically ill or not^11–15,27–30^. Baseline demographics and comorbidities of patients included in the studies are shown in Table 2.

**Table 2:** Baseline Demographics of patients with COVID-19.

Author	AGE (median)	Male gender, n (%)	Hypertension, n (%)	Diabetes, n (%)	Cardiovascular disease, n (%)	COPD, n (%)
Ruan et al.^22^	57.7	102/150 (68)	53/150 (35)	26/150 (17)	14/150 (9)	3/150 (2)
Deng et al.^23^	54.1	124/225 (55)	59/225 (26)	27/225 (12)	18/225 (8)	25/225 (11)
Huang et al.^14^	49	30/41 (73)	6/41 (15)	8/41 (20)	6/41 (15)	1/41 (2)
Wu et al.^26^	52.4	128/201 (64)	38/201 (19)	22/201 (11)	8/201 (4)	NR
Wang et al.^11^	56	75/138 (54)	43/138 (31)	14/138 (10)	21/138 (15)	4/138 (3)
Zhou et al.^24^	56	119/191 (62)	57/191 (30)	36/191 (19)	15/191 (8)	6/191 (3)
Chen et al.^25^	62	171/274 (62)	93/274 (34)	47/274 (17)	22/274 (8)	19/274 (7)
Chen et al.^12^	58.9	84/150 (56)	50/150 (33)	20/150 (13)	9/150 (6)	NR
Deng et al.^13^	65	57/112 (51)	36/112 (32)	19/112 (17)	15/112 (13)	4/112 (4)
Guo et al.^20^	58.5	91/187 (49)	62/187 (33)	28/187 (15)	21/187 (11)	4/187 (2)
Peng et al.^15^	62	53/112 (47)	92/112 (82)	24/112 (21)	62/112 (55)	NR
Shi et al.^21^	64	205/416 (49)	129/416 (31)	58/416 (14)	46/416 (11)	12/416 (3)
Han et al.^29^	58	26/75 (35)	NR	NR	NR	NR
Wang et al.^28^	NR	48/110 (44)	23/110 (21)	15/110 (14)	NR	6/110 (5)
Yan et al.^26^	69.4	33/48 (69)	24/48 (50)	48/193 (25)	13/48 (27)	4/48 (8)
Zheng et al.^30^	49.4	51/99 (52)	NR	2.5/41 (6)	NR	NR

*NR* not reported.

### Troponin

**Figure 2:**
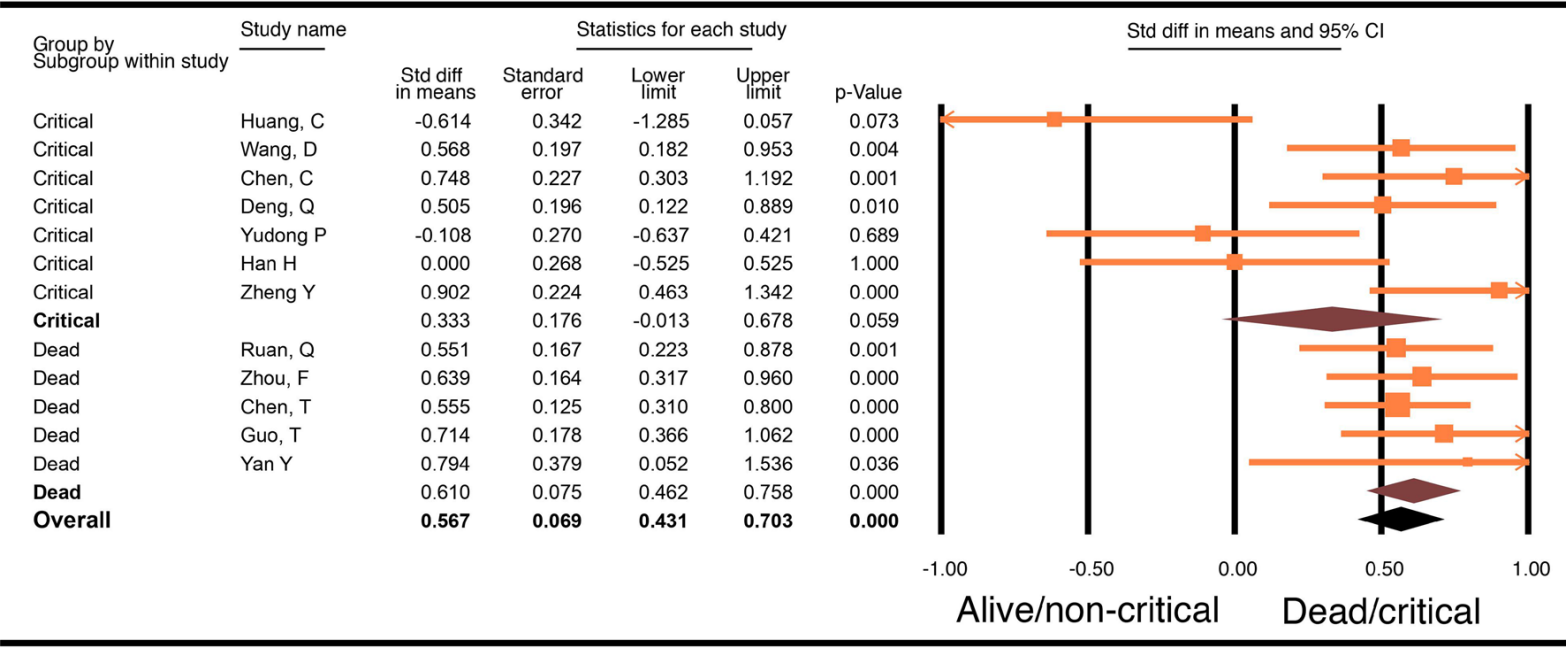
Forest plot of WMD in troponin between alive and/or not critically ill patients and dead and/or critically ill patients with COVID-19.

Twelve studies with a total of 1,715 patients were included for this analysis. Seven of the studies compared critically ill versus not critically ill patients, and the other 5 studies compared levels in dead versus alive patients. Results showed that patients who died or were critically ill had significantly higher troponin levels compared to patients who were alive or were not critically ill (WMD 0.57, 95% CI 0.43–0.70, p < 0.001). Sub-group analysis of the studies evaluating patients who died compared to patients who were alive showed that there was a significantly higher troponin level in those who died (WMD 0.61, 95% CI 0.46–0.76, p < 0.001). On the other hand, meta-analysis of studies comparing troponin levels in patients who were critically ill to those in patients who were not critically ill showed no difference in troponin levels (WMD 0.28, 95% CI − 0.14–0.69, p = 0.059, Fig. 2). The difference between these 2 groups of studies, i.e., studies that compared dead and alive and those that compared critically ill and not critically ill patients, was not significantly different (p 0.14).

The test for heterogeneity for the analysis of the 12 studies was an I^2^ of 54.5; therefore, a multivariate meta-regression analysis was performed with age, male gender, diabetes, hypertension, and cardiovascular disease. This analysis showed that hypertension (p = 0.027, Supplemental Fig. 2) and cardiovascular disease (p = 0.001), (Supplemental Fig. 3), contributed to the differences among studies in the reported troponin levels between the compared groups (R = 1). Specifically, with increasing incidence of hypertension, there was a higher WMD in the troponin levels in patients who were dead or critically ill compared to levels in patients who were alive or not critically ill. The incidence of cardiovascular disease in the studies, on the other hand, was inversely related to the WMD of troponin between the groups.

Risk of death based on biomarker proven cardiac injury: we included 4 studies that reported a hazard ratio for death or event rates based on cardiac injury. Of these studies, 1 study used CK-MB without providing a definition^27^. One study^16^ used troponin T and defined cardiac injury as troponin T levels above the 99th percentile upper reference limit. Troponin I levels were used in two studies, and both studies defined cardiac injury as troponin I levels above the 99th percentile upper reference limit^21,24^.

For the purpose of the meta-analysis, we extracted adjusted effect sizes, when available. In studies that did not report adjusted effect sizes, unadjusted OR was extracted. One study reported number of deaths and total sample size in each group, which was used in the analysis. Our analysis showed that cardiac injury was independently associated with significantly increased odds of mortality (OR 6.641, 95% CI 1.26–35.1, p = 0.03).

### BNP

**Figure 3:**
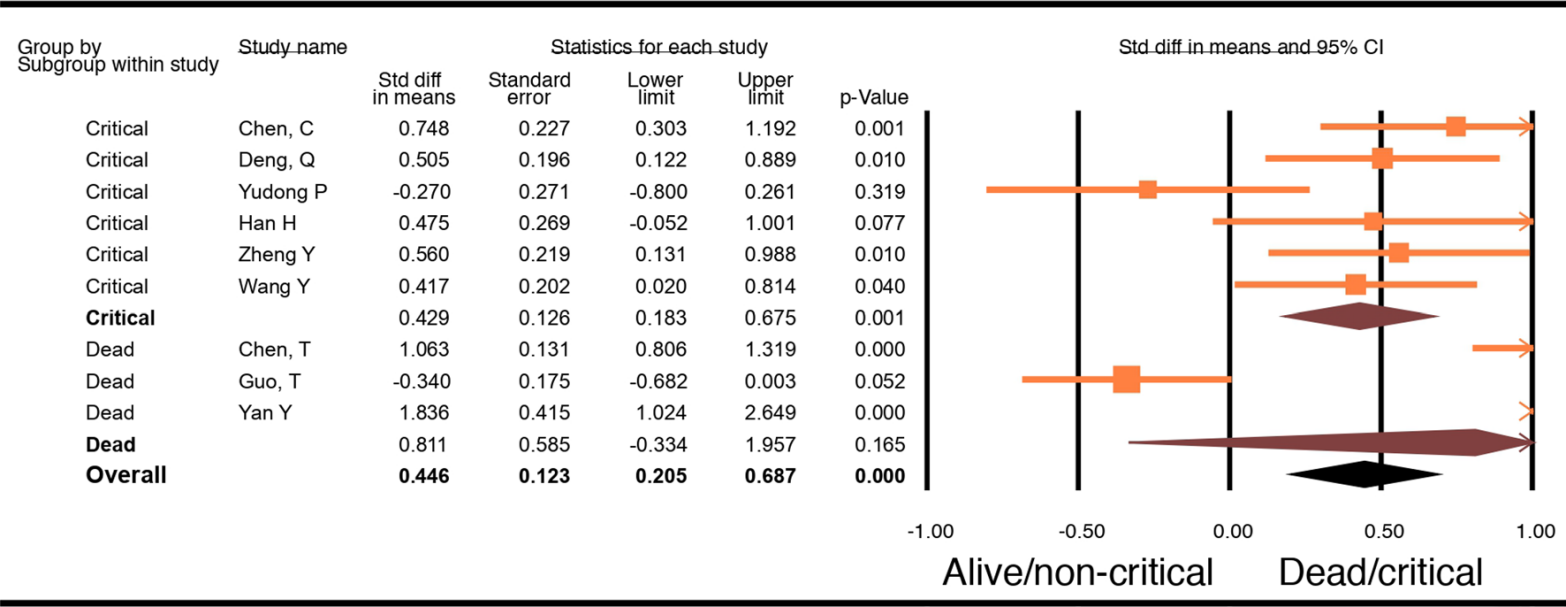
Forest plot of WMD in BNP between alive and/or not critically ill patients and dead and/or critically ill patients with COVID-19.

There were 9 studies with 1305 patients that reported NT-proBNP. Six studies compared critically ill versus not critically ill patients, and the other 3 studies compared results from dead and alive patients. Analysis of these 9 studies did show a significant difference in BNP levels in patients who died or were critically ill compared to levels in those who were alive or were not critically ill (WMD0.45, 95% CI − 0.21–0.69, p < 0.001). Sub-group analysis showed that there was no significant difference in patients who died compared to patients who were alive (WMD 0.81, 95% CI − 0.33–1.96, p = 0.17); however, there was a significant difference seen in patients who were critically ill and those who were not (WMD 0.43, 95% CI − 0.18–0.68, p = 0.001) (Fig. 3).

### Creatinine kinase (CK)

Eight studies with a total of 1,066 patients were included in this analysis. Four of the studies compared critically ill versus not critically ill patients, and the other 4 studies compared CK levels in dead patients versus alive patients. There was no significant difference in the CK levels in patients who died or were critically ill and those in patients who were alive or were not critically ill (WMD 0.21, 95% CI − 0.05–0.47, p = 0.12). Sub-group analysis showed that there was a significantly higher CK level in patients who died (WMD 0.79, 95% CI 0.25–1.33, p = 0.004) compared to patients who survived, whereas the patients who were critically ill did not have significantly higher CK levels compared to the patients who were not critically ill (WMD 0.04, 95% CI − 0.26–0.33, p = 0.82). (Supplemental Fig. 1) The difference between these 2 groups of studies (studies comparing dead versus alive and those that compared critically ill versus not critically ill patients) was not significantly different.

### D-dimer

Nine studies with a total of 1214 patients evaluated D-dimer levels in COVID-19 patients. Six studies compared critically ill versus not critically ill patients, and the other three compared levels from dead patients versus alive patients. Analysis of these nine studies showed that patients who died or were critically ill had significantly higher D-dimer levels compared to patients who were alive or were not critically ill (WMD OR 0.66, 95% CI 0.47–0.85, p < 0.001). Sub-group analysis of studies evaluating patients who died compared to patients who were alive showed that there was a significantly higher D-dimer level in those who died (WMD 0.57, 95% CI 0.32–0.82, p < 0.001). Similarly, critically ill patients had a significantly higher D-dimer level compared to the patients who were not critically ill (WMD 0.79, 95% CI 0.49–1.08, p < 0.001). (Supplemental Fig. 4).

### LDH

Nine studies with a total of 1267 patients evaluated LDH levels in COVID-19 patients. Five of the studies compared critically ill versus not critically ill patients, and the other four compared levels from dead patients versus alive patients. Analysis of these eight studies showed that patients who died or were critically ill had significantly higher LDH levels compared to patients who were alive or were not critically ill (WMD 0.58, 95% CI 0.39–0.74, p < 0.001). Sub-group analysis showed that there was a significantly higher LDH level in those who died (WMD 1.41, 95% CI − 0.41–2.41, p = 0.006) compared to those who were alive, and that critically ill patients had significantly higher LDH levels compared to patients who were not critically ill (WMD 0.54, 95% CI 0.36–0.72, p < 0.001). The difference between these two groups of studies, dead versus alive and studies that compared critically ill versus not critically ill patients, was not significantly different. (Supplemental Fig. 5).

## Discussion

**Figure 4:**
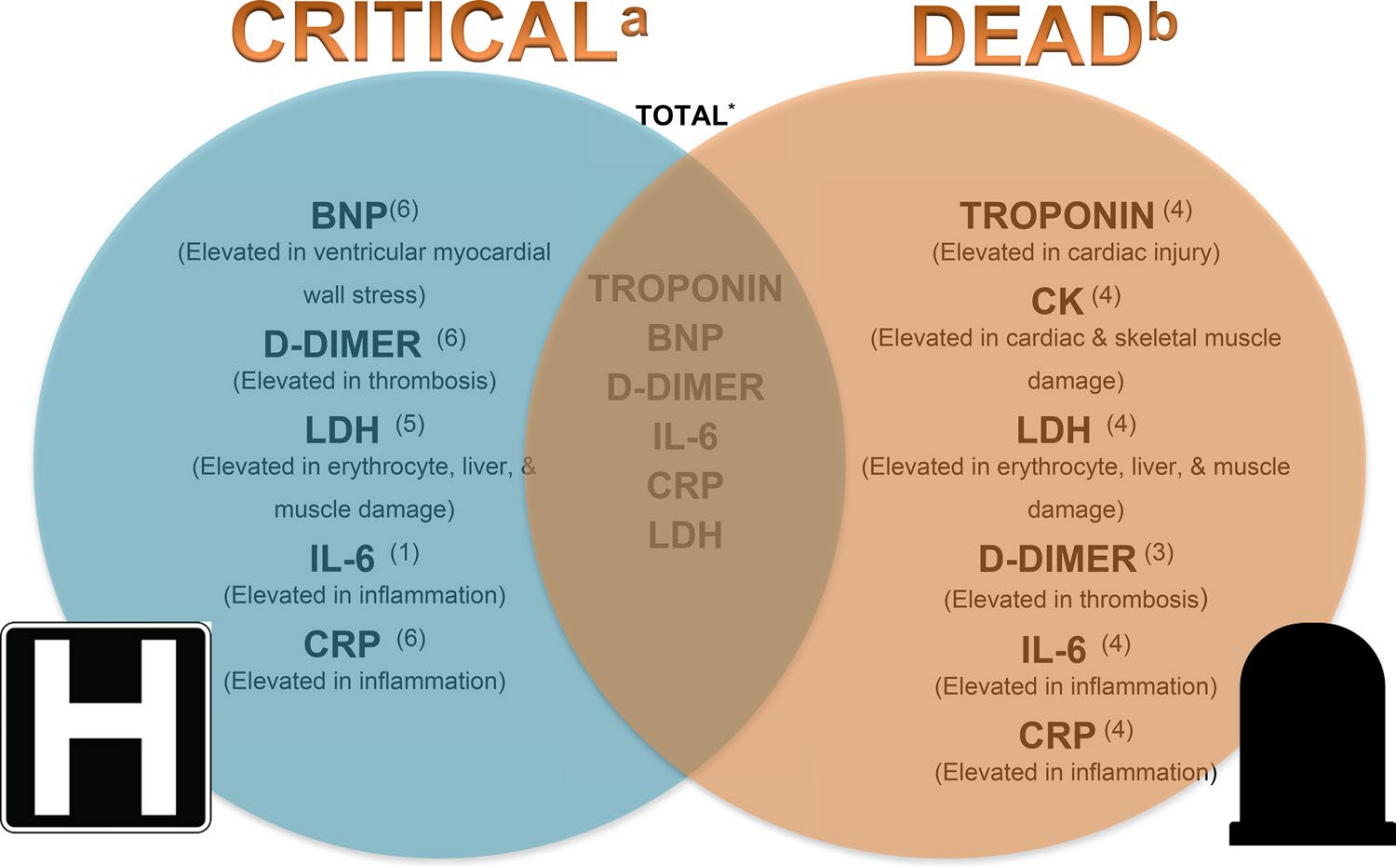
Biomarkers in COVID-19 patients. ^a^Significant difference seen in biomarkers in COVID-19 patients who were critically ill. ^b^Significant difference seen in biomarkers in COVID-19 patients who died. *Significant difference seen in biomarkers in COVID-19 patients who died and/or were critically ill**.** Superscripts: total number of articles in each group. Biomarkers studied: Troponin, BNP, CK, CRP, LDH, D-dimer, IL-6.

This meta-analysis summarizes current evidence regarding biomarkers as prognosticators of outcomes in Covid-19 infection and outlines the utility of cardiac biomarkers to predict severe illness and/or death and is unique in that it is the only meta-analysis within the studied time frame that analyzed troponin in critically ill and dead patients as separate subgroups. Our study demonstrates that there exists a statistically significant association between disease severity and cardiac injury. Our analysis showed that troponin and BNP levels were higher among patients who died or were critically ill compared to those in patients who survived or were not critically ill (Fig. 4). When analyzed by sub-groups, we found that levels of troponin and CK were significantly elevated in patients with a fatal outcome compared to those who did not die, but were not significantly different in patients who were critically ill and those who were not (Fig. 4). Prior meta-analyses showed an association between increase in troponin levels in regards to mortality or severe disease (i.e., patients requiring mechanical ventilation, ICU admission, or those who died); however, a sub-group analysis of the biomarkers was not performed^31,32^. The results of our meta-analysis are also different from the ones published by Santoso et.al in which they found increased troponin levels in patients who died as well as in patients who were admitted to the ICU or had severe COVID-19 infection. Additionally, these studies only included troponin as the cardiac marker in their analysis as a prognostic indicator^31–33^. Our sub-group analysis also found that BNP levels were significantly different in critically ill patients compared to those who were not critically ill.

While pre-existing cardiac co-morbidities increase the risk of Acute COVID-19 Cardiovascular Syndrome (ACoVCS)^34,35^, recent evidence shows that SARS-CoV-2 can cause acute myocarditis-like illness in an otherwise healthy patient^36^. The mechanism of cardiac injury caused by SARS-CoV-2 is not yet understood, but possible mechanisms include cytokine storm as a result of dysfunctional and uncontrolled immunological response, evidenced by increased levels of d-dimer, IL-6, CRP and LDH^14,37^, and primary cardiac injury caused by SARS-CoV-2. The first case of acute cardiac injury directly linked to myocardial localization of SARS-CoV-2, demonstrated by endomyocardial biopsy, was reported by Tavazzi et al.^38^ The viral envelope of SARS-CoV-2 is studded with glycoproteins called Spike proteins composed of two subunits. Subunit S1 has affinity for ACE2 receptors on the cell surface and Subunit S2 fuses with the cell membrane; acting together, these two proteins aid in the endocytosis of the viral particle^39,40^. Cardiac tissue has a significantly higher expression of ACE2 receptors and is therefore prone to direct cardiac injury in patients with COVID-19^41,42^.

A recent study done by Liu et al. showed that increased levels of Angiotensin-2 (AT-2) in the plasma of patients infected with COVID-19 showed a linear association with the severity of the disease^43^. SARS-CoV-2 reduces the expression of ACE2 due to internalization as shown in a study by Oudit et al., which demonstrated a marked decrease in ACE2 expression in the myocardium of mice infected with SARS-CoV-2^44^. ACE2 is responsible for the degradation of AT-2 into Ang 1–7, which is cardioprotective and has anti-inflammatory and anti-fibrotic properties^45^. The loss of ACE2 shifts the balance towards an accumulation of AT-2, which has inflammatory and pro-thrombotic properties. AT-2 promotes vasoconstriction and increases the levels of Plasminogen Activator Inhibitor-1, which plays an active role in thrombogenesis and myocardial infarction^45^. This shift in RAAS axis towards AT-2 could possibly explain high D-dimer levels, a biomarker that showed clearly increased levels in both critically ill patients and patients who died compared to levels in not critically ill and alive patients in our meta-analysis (Fig. 4).

Surprisingly, among the cardiac biomarkers (troponin, CK, and BNP) analyzed in this meta-analysis, only BNP had levels significantly higher in critically ill patients compared to levels in patients who were not critically ill, the levels of CK and troponin were significantly higher in patients who died compared to the levels in those who survived (Fig. 4). Surprisingly, based on our meta-analysis, BNP levels did not differ in patients who died and those who lived. In addition to ventricular stretch, BNP is a known marker for many other conditions, including lung disease, pulmonary hypertension, kidney dysfunction, and excessive cortisol levels. COVID-19 infection causes an assortment of these conditions in the different groups of patients, effectively neutralizing the differences between the groups. Troponin and BNP were both higher in the combined analysis of critically ill and/or dead COVID-19 patients compared to not critically ill and/or alive patients. These results suggest that while there could be minimal changes in troponin in critically ill patients due possibly to non-cardiac reasons, significant elevation in COVID-19 patients may be an indication of direct myocardial injury, which in turn increases the risk of death, thereby making it a crucial tool in predicting death. Unfortunately, not many studies reported the MB isofraction of CK. In addition, the inflammatory biomarkers (IL-6 and CRP; see Supplementary Figs. 6 and 7) and D-dimer levels from the same studies were significantly increased in the critically ill patients and the patients who died compared to levels in the control population, suggesting that it might be difficult to use these biomarkers to differentiate patients who are just critically ill from those at risk of dying. However, the difference in IL-6 (p 0.04) and CRP (p 0.04) levels between patients who died compared to levels in patients who survived was significantly higher than in patients who were critically ill and those who were not (Fig. 4). Finally, LDH was significantly higher in both critically ill patients and patients who died compared to those who had mild illness and those who survived.

## Limitations

Our study has several limitations. First, the critical and not critical groups included in our analysis are heterogenous, i.e., we defined critical as patients who required ICU admission as well as those who developed severe lung injury and ARDS. Similarly, the criteria for ICU admissions may differ from one hospital to another. Second, most studies were conducted in and published from China and therefore the generalizability of this information to other parts of the world and ethnicities is limited. The absence of details regarding the timing of measurement of biomarkers during the hospital stay limits the use of the results as an aid to the ‘predictability’ of death or critical illness. Last, the studies included in the meta-analysis evaluating the risk of mortality with cardiac injury varied in their definitions of cardiac injury.

## Supplementary Information

Supplementary Figures.

## Data Availability

All data generated or analyzed during this study are included in this published article (and its Supplementary Information files).

## Abbreviations

COVID-19: Coronavirus 2019
WMD: Weighted mean differences
CI: 95% Confidence interval
OR: Odds ratio
BNP: Brain natriuretic peptide
CK: Creatine kinase
LDH: Lactate dehydrogenase
CRP: C-reactive protein
IL-6: Interleukin 6
SARS-CoV-2: Severe acute respiratory syndrome coronavirus-2
