# Supplementary material for: Prognostic value of cardiac biomarkers in COVID-19 infection
Source: Sci Rep. 2021 Mar 2;11:4930. doi: 10.1038/s41598-021-84643-6 (PMC7925599; doi:10.1038/s41598-021-84643-6)
Supplement: Supplementary file 1 — Supplementary Figures. [file 41598_2021_84643_MOESM1_ESM.pdf]

Prognostic value of Cardiac Biomarkers in COVID-19 Infection.

Aakash Sheth*, MD, Malak Modi*, MD, Desiree' Dawson*, MD, Paari Dominic, MD

*Equal contributors

Supplemental Figure Legends:

Supplemental Figure 1: Forest plot of WMD in CK between alive and/or not critically ill patients and dead and/or critically ill patients with COVID-19.
Supplemental Figure 2: Relationship between HTN and WMD in troponin levels in individual studies in a multivariate meta-regression model.
Supplemental Figure 3: Relationship between CVD and WMD in troponin levels in individual studies in a multivariate meta-regression model.
Supplemental Figure 4: Forest plot of WMD in D-dimer between alive and/or not critically ill patients and dead and/or critically ill patients with COVID-19.
Supplemental Figure 5: Forest plot of WMD in LDH between alive and/or not critically ill patients and dead and/or critically ill patients with COVID-19.
Supplemental Figure 6: Forest plot of WMD in IL-6 between alive and/or not critically ill patients and dead and/or critically ill patients with COVID-19.
Supplemental Figure 7: Forest plot of WMD in CRP between alive and/or not critically ill patients and dead and/or critically ill patients with COVID-19.
Supplemental Figure 8: Funnel plot for publication bias for studies reporting Troponin.
Supplemental Figure 9: Funnel plot for publication bias for studies reporting CK.
Supplemental Figure 10: Funnel plot for publication bias for studies reporting BNP.
Supplemental Figure 11: Funnel plot for publication bias for studies reporting D-dimer.
Supplemental Figure 12: Funnel plot for publication bias for studies reporting LDH.
Supplemental Figure 13: Funnel plot for publication bias for studies reporting IL-6.
Supplemental Figure 14: Funnel plot for publication bias for studies reporting CRP.

Group by Subgroup within study	Study name	Std diff in means	Standard error	Lower limit	Upper limit	p-Value
Critical	Huang, C	-0.345	0.338	-1.007	0.317	0.307
Critical	Wang, D	0.342	0.195	-0.040	0.724	0.079
Critical	Deng, Q	-0.159	0.193	-0.538	0.219	0.409
Critical	Yudong P	0.146	0.270	-0.384	0.676	0.589
**Critical**		0.035	0.152	-0.264	0.333	0.821
Dead	Ruan, Q	0.096	0.164	-0.226	0.417	0.559
Dead	Zhou, F	0.537	0.163	0.218	0.857	0.001
Dead	Chen, T	0.737	0.127	0.489	0.985	0.000
Dead	Yan Y	2.387	0.443	1.519	3.255	0.000
**Dead**		0.788	0.276	0.247	1.329	0.004
**Overall**		**0.210**	**0.133**	**-0.051**	**0.472**	**0.115**

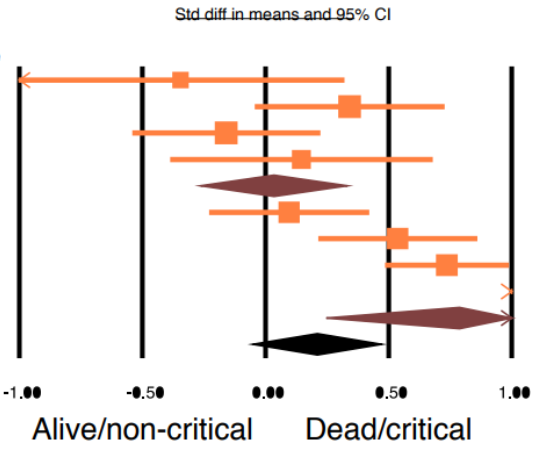

Supplemental Figure 1. Forest plot of WMD in CK between alive and/or not critically ill patients and dead and/or critically ill patients with COVID-19.

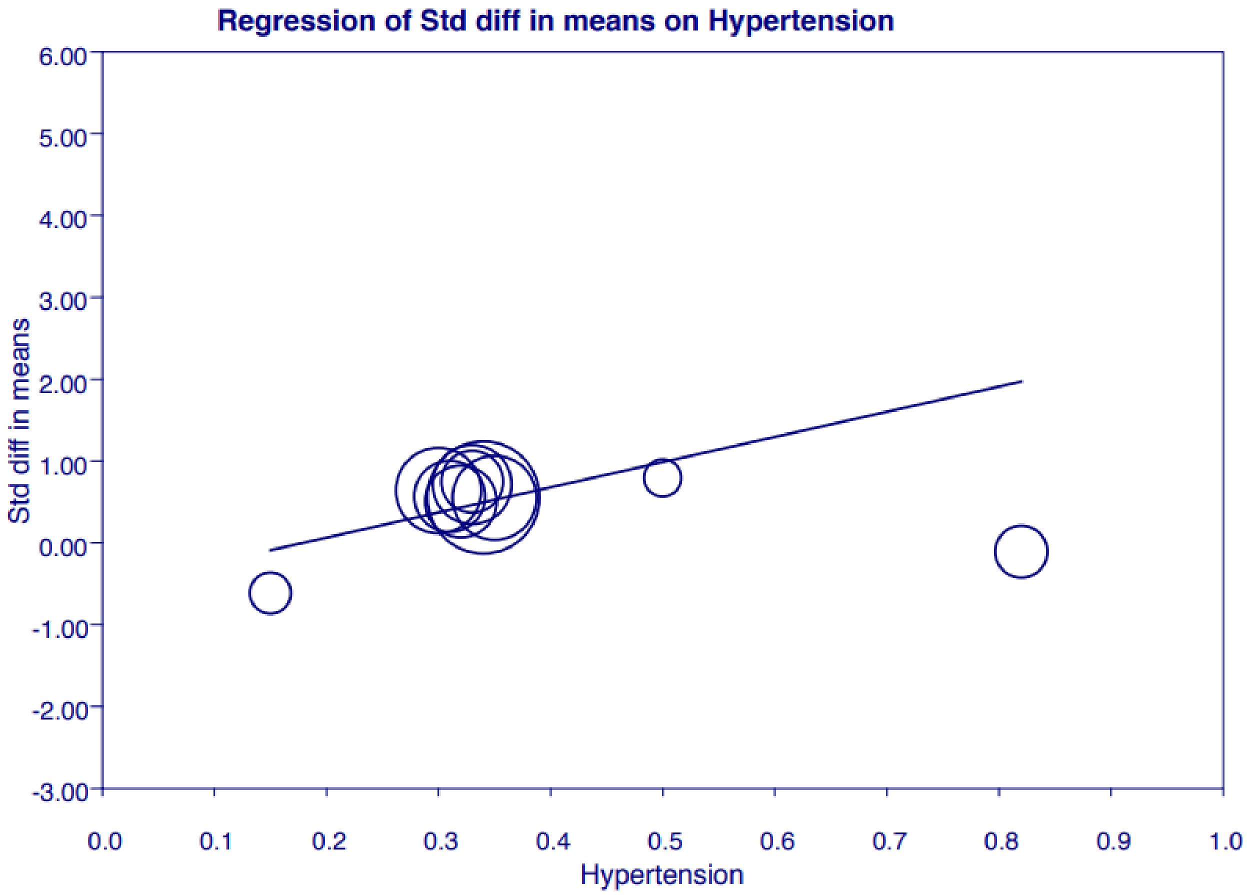

Supplemental Figure 2. Relationship between HTN and WMD in troponin levels in individual studies in a multivariate meta-regression model.

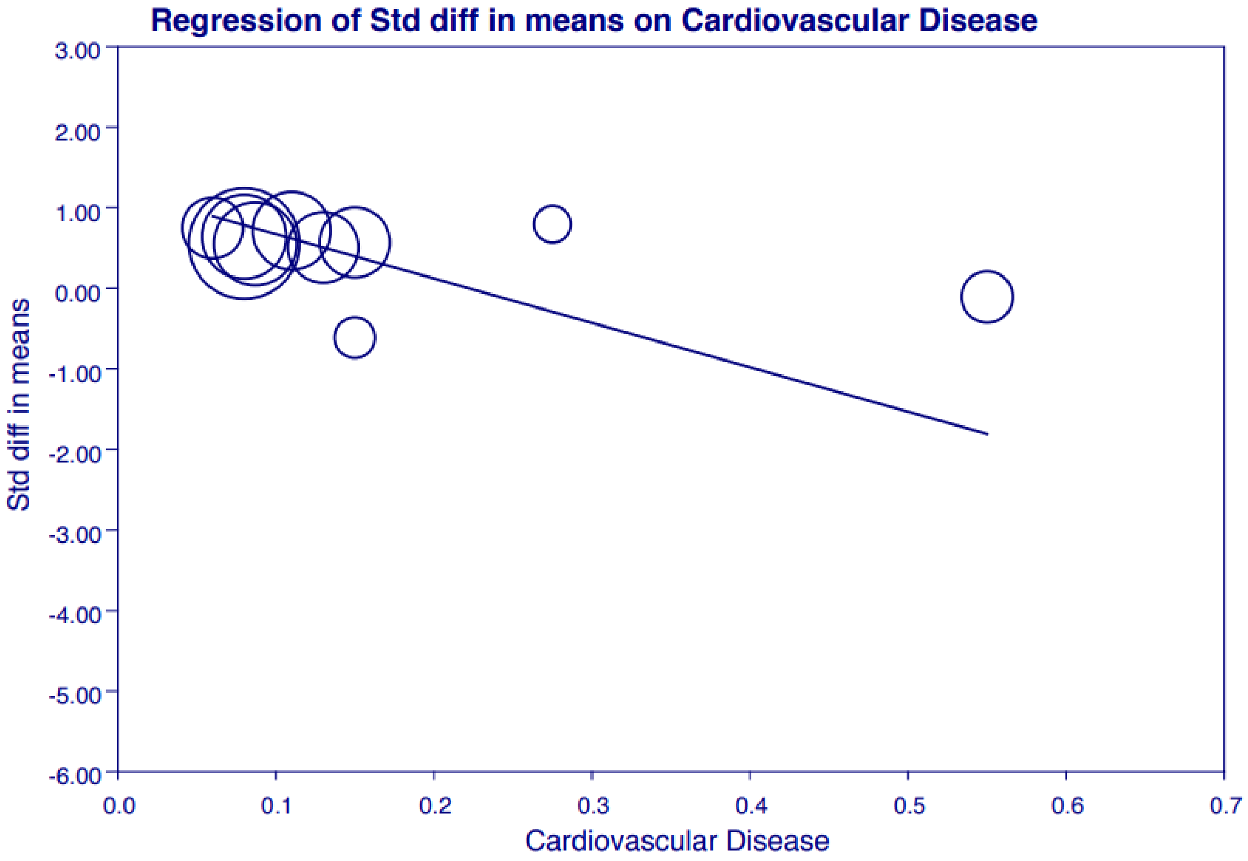

Supplemental Figure 3. Relationship between CVD and WMD in troponin levels in individual studies in a multivariate meta-regression model.

Group by Subgroup within study	Study name	Std diff in means	Standard error	Lower limit	Upper limit	p-Value
		Statistics for each study				
Critical	Huang, C	1.021	0.354	0.327	1.715	0.004
Critical	Wu, C	0.478	0.145	0.194	0.762	0.001
Critical	Wang, D	0.652	0.198	0.264	1.040	0.001
Critical	Deng, Q	0.505	0.196	0.122	0.889	0.010
Critical	Zheng Y	0.810	0.222	0.374	1.246	0.000
Critical	Wang Y	1.446	0.223	1.009	1.883	0.000
**Critical**		0.786	0.152	0.488	1.083	0.000
Dead	Zhou, F	0.639	0.164	0.317	0.960	0.000
Dead	Chen, T	0.426	0.124	0.183	0.670	0.001
Dead	Yan Y	1.041	0.385	0.286	1.795	0.007
**Dead**		0.568	0.127	0.319	0.817	0.000
**Overall**		**0.658**	**0.097**	**0.467**	**0.849**	**0.000**

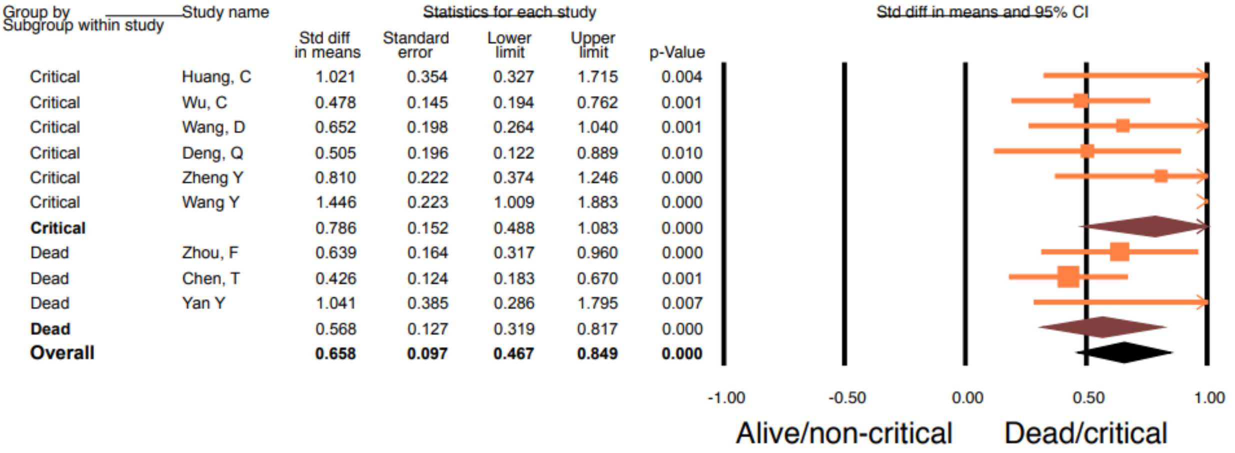

Supplemental Figure 4. Forest plot of WMD in D-dimer between alive and/or not critically ill patients and dead and/or critically ill patients with COVID-19.

Group by Subgroup within study	Study name	Statistics for each study				
		Std diff in means	Standard error	Lower limit	Upper limit	p-Value
Critical	Huang, C	1.015	0.354	0.321	1.708	0.004
Critical	Wu, C	0.478	0.145	0.194	0.762	0.001
Critical	Wang, D	0.652	0.198	0.264	1.040	0.001
Critical	Deng, Q	0.505	0.196	0.122	0.889	0.010
Critical	Yudong P	0.333	0.271	-0.198	0.864	0.219
**Critical**		0.540	0.091	0.361	0.718	0.000
Dead	Ruan, Q	0.289	0.165	-0.034	0.612	0.079
Dead	Zhou, F	0.639	0.164	0.317	0.960	0.000
Dead	Chen, T	2.013	0.150	1.719	2.307	0.000
Dead	Yan Y	2.961	0.478	2.025	3.898	0.000
**Dead**		1.411	0.509	0.414	2.408	0.006
**Overall**		**0.567**	**0.090**	**0.391**	**0.742**	**0.000**

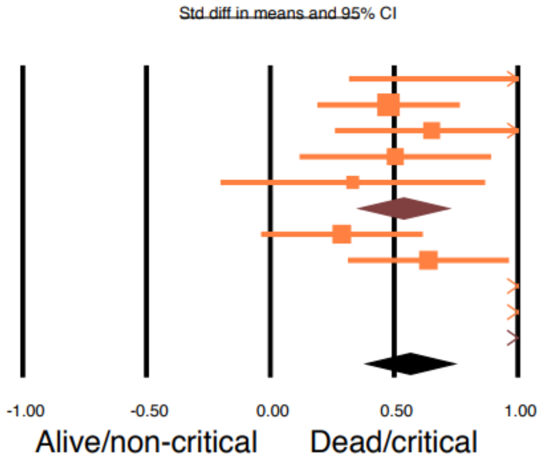

Supplemental Figure 5. Forest plot of WMD in LDH between alive and/or not critically ill patients and dead and/or critically ill patients with COVID-19.

Group by Subgroup within study	Study name	Statistics for each study				
		Std diff in means	Standard error	Lower limit	Upper limit	p-Value
Critical	Wu, C	0.313	0.144	0.031	0.595	0.030
**Critical**		0.313	0.144	0.031	0.595	0.030
Dead	Ruan, Q	0.551	0.167	0.223	0.878	0.001
Dead	Zhou, F	0.639	0.164	0.317	0.960	0.000
Dead	Chen, T	1.086	0.131	0.829	1.343	0.000
Dead	Yan Y	1.317	0.393	0.546	2.088	0.001
**Dead**		0.837	0.165	0.514	1.161	0.000
**Overall**		0.539	0.108	0.326	0.752	0.000

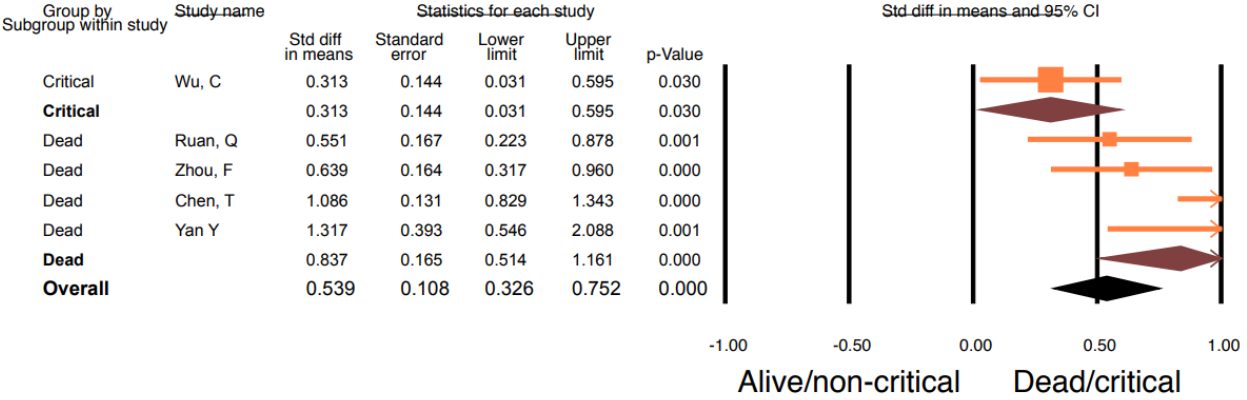

Supplemental Figure 6. Forest plot of WMD in IL-6 between alive and/or not critically ill patients and dead and/or critically ill patients with COVID-19.

Group by Subgroup within study	Study name	Std diff in means	Standard error	Lower limit	Upper limit	p-Value
Critical	Wu, C	0.478	0.145	0.194	0.762	0.001
Critical	Chen, C	0.748	0.227	0.303	1.192	0.001
Critical	Deng, Q	0.505	0.196	0.122	0.889	0.010
Critical	Yudong P	0.913	0.277	0.370	1.456	0.001
Critical	Zheng Y	1.177	0.231	0.725	1.629	0.000
Critical	Wang Y	3.888	0.330	3.241	4.534	0.000
**Critical**		1.254	0.394	0.483	2.026	0.001
Dead	Ruan, Q	0.551	0.167	0.223	0.878	0.001
Dead	Deng, Y	1.502	0.151	1.206	1.798	0.000
Dead	Chen, T	1.600	0.140	1.324	1.875	0.000
Dead	Yan Y	2.885	0.473	1.959	3.812	0.000
**Dead**		1.524	0.330	0.877	2.171	0.000
**Overall**		**1.412**	**0.253**	**0.917**	**1.908**	**0.000**

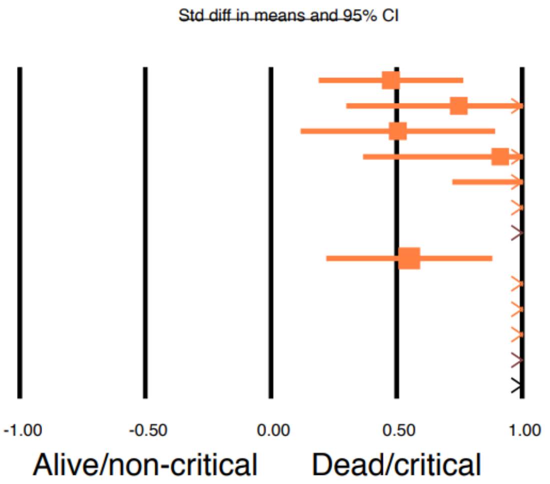

Supplemental Figure 7. Forest plot of WMD in CRP between alive and/or not critically ill patients and dead and/or critically ill patients with COVID-19.

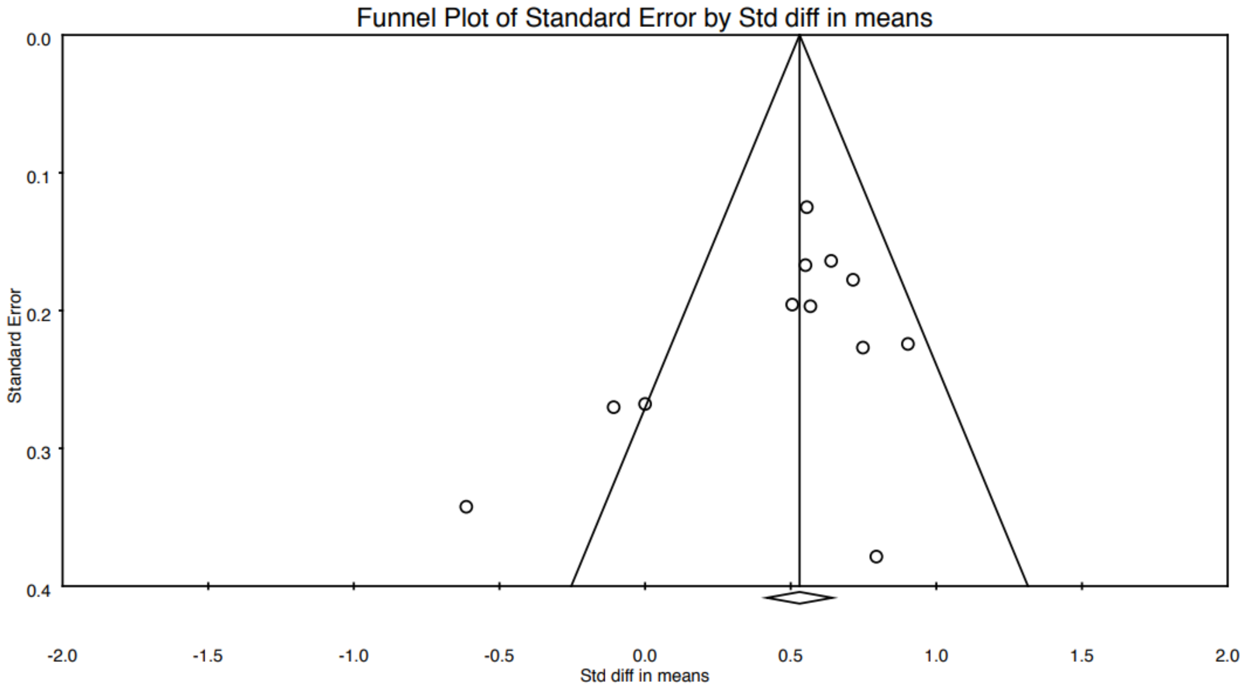

Supplemental Figure 8: Funnel plot for publication bias for studies reporting Troponin

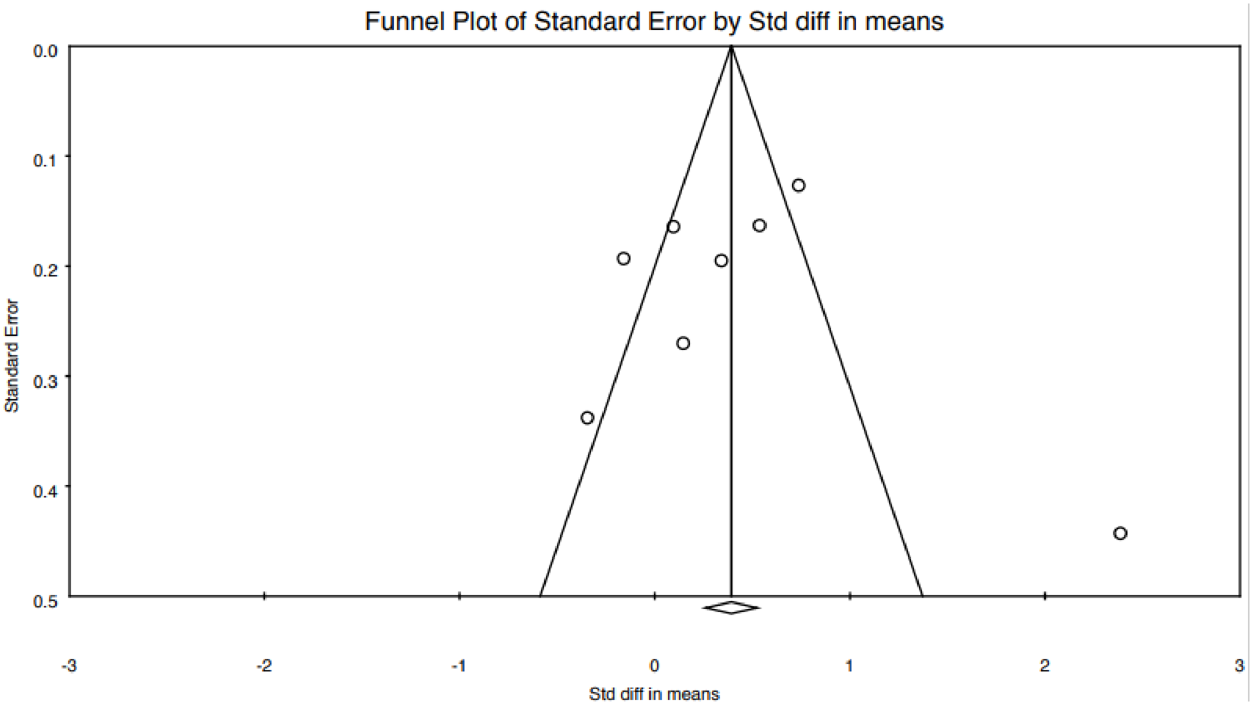

Supplemental Figure 9: Funnel plot for publication bias for studies reporting CK

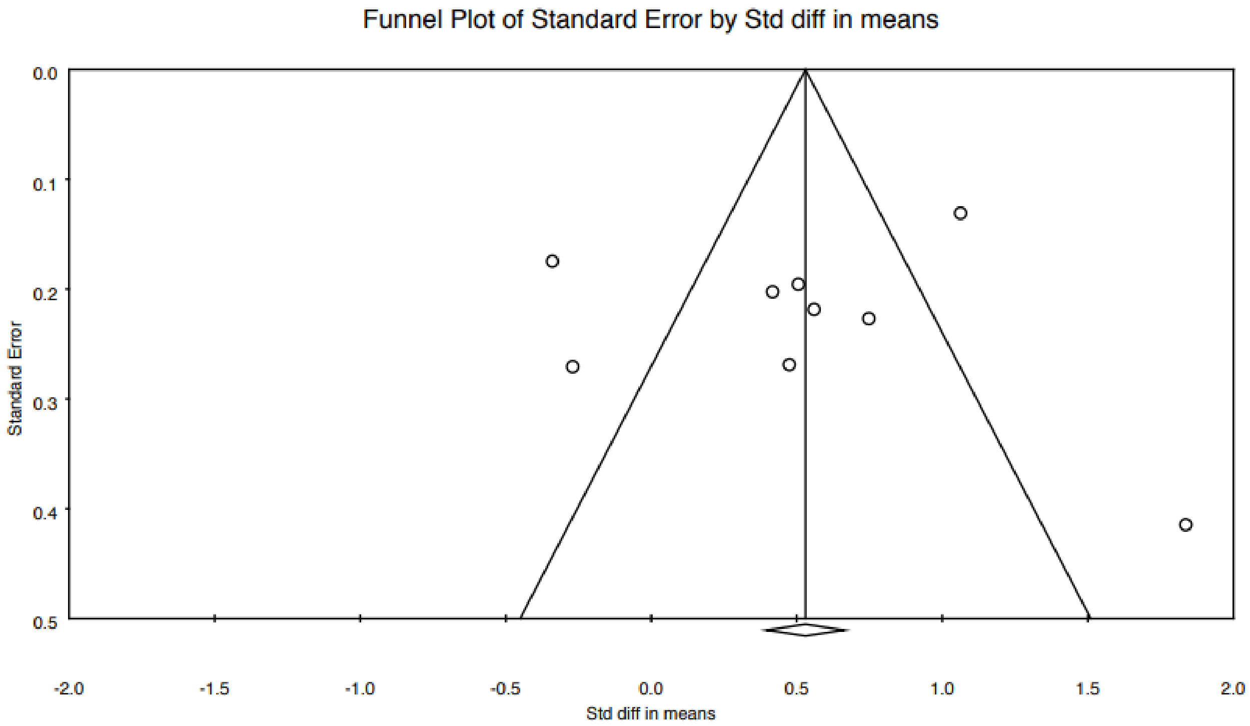

Supplemental Figure 10: Funnel plot for publication bias for studies reporting BNP

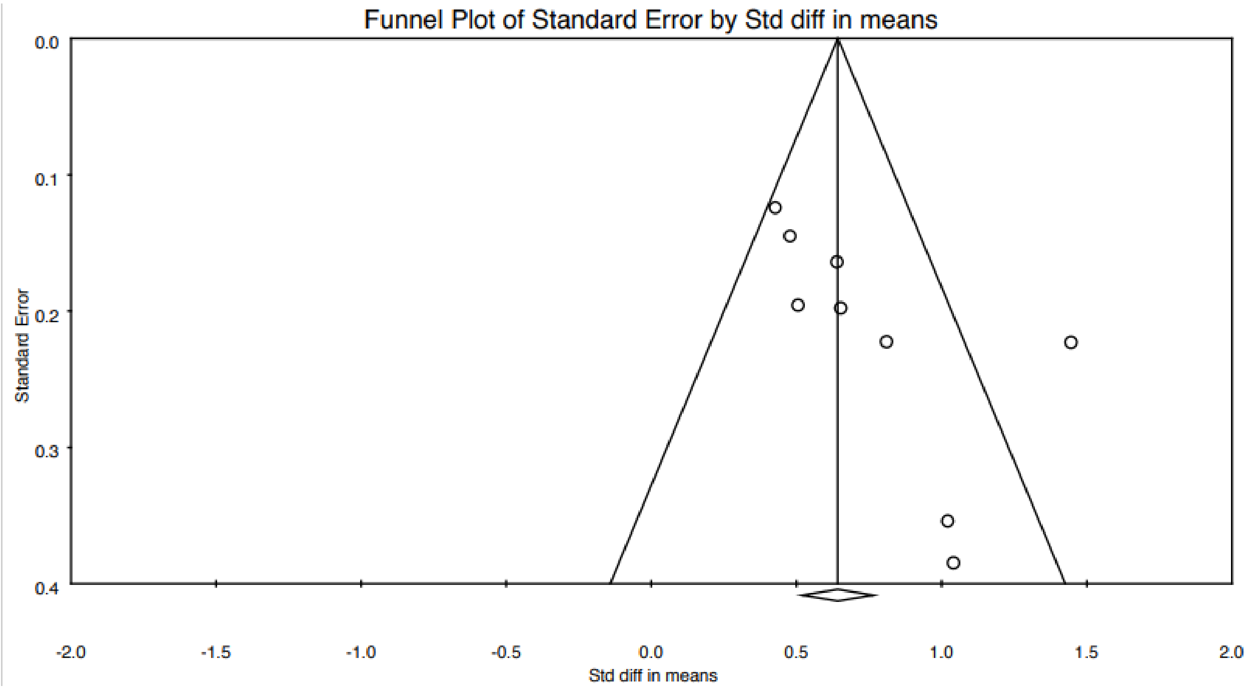

Supplemental Figure 11: Funnel plot for publication bias for studies reporting D-dimer

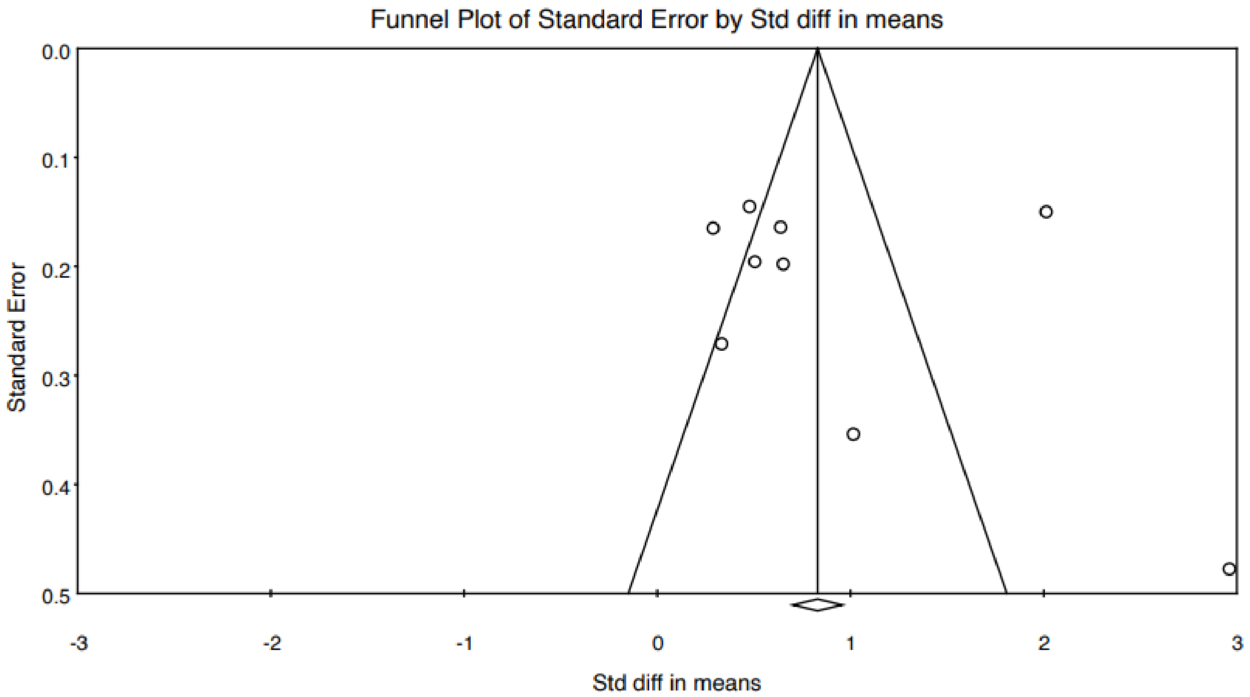

Supplemental Figure 12: Funnel plot for publication bias for studies reporting LDH

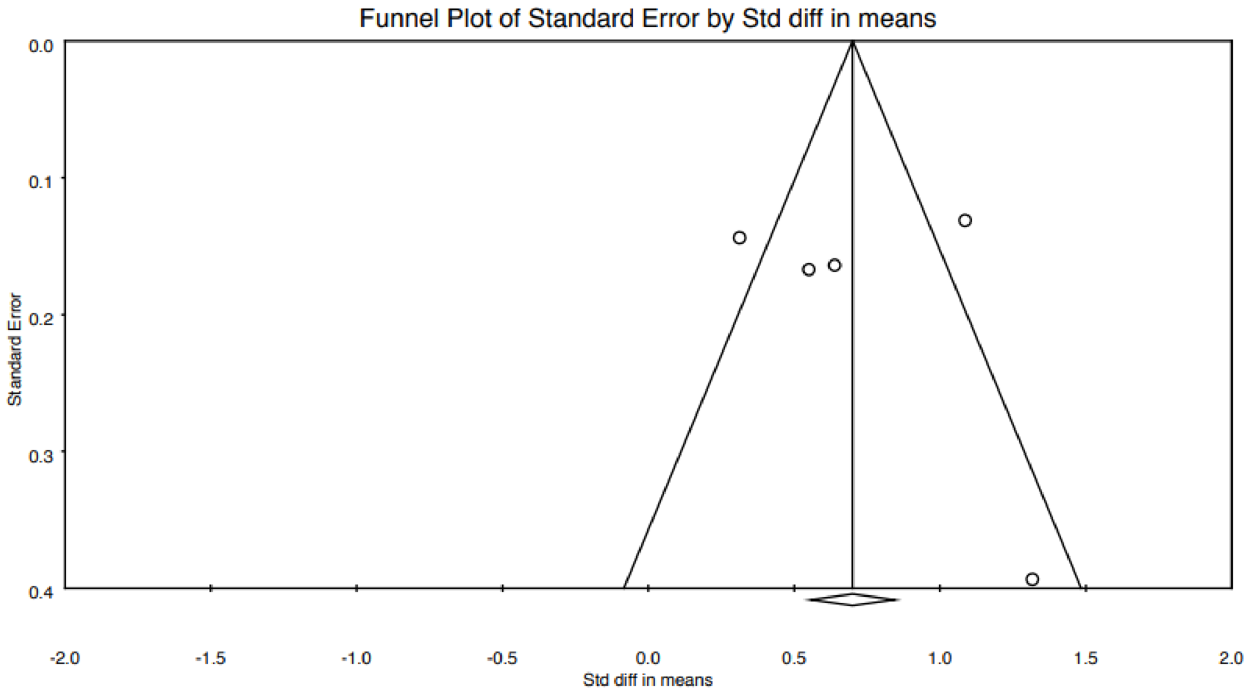

Supplemental Figure 13: Funnel plot for publication bias for studies reporting IL-6

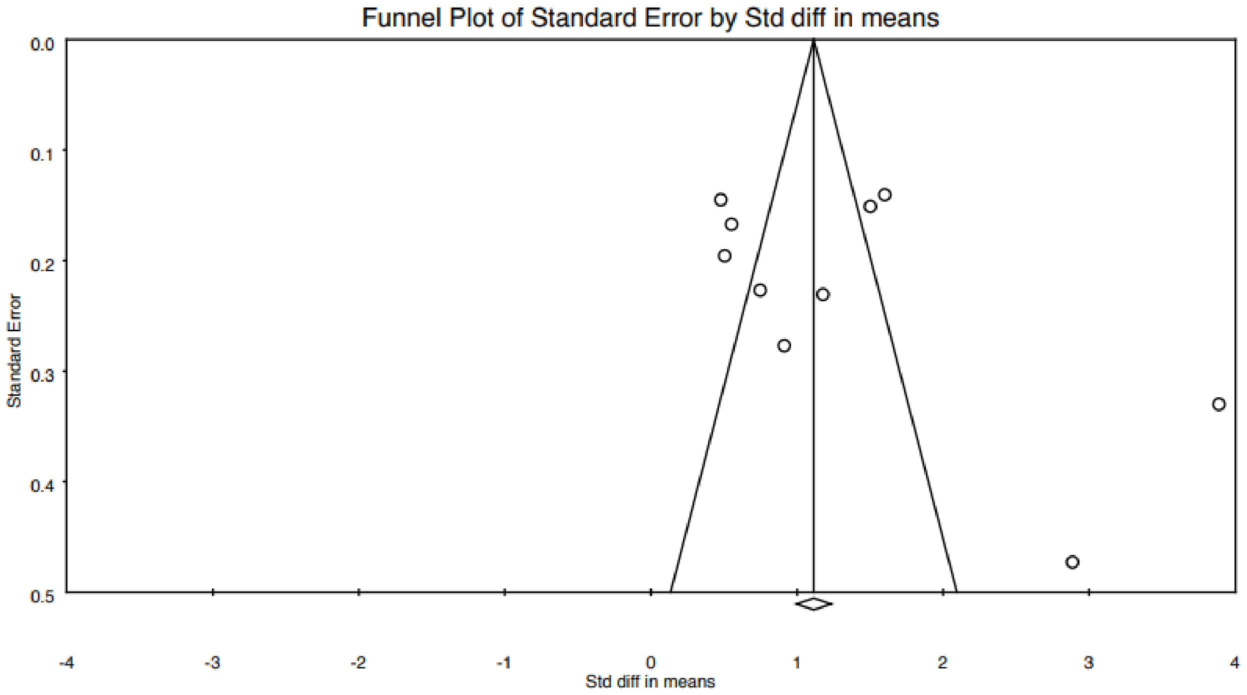

Supplemental Figure 14: Funnel plot for publication bias for studies reporting CRP
